# CEP55 as a promising biomarker and therapeutic target on gallbladder cancer

**DOI:** 10.3389/fonc.2023.1156177

**Published:** 2023-05-18

**Authors:** Maotuan Huang, Fuxiu Zhong, Mingyuan Chen, Lingju Hong, Weihong Chen, Xiahenazi Abudukeremu, Feifei She, Yanling Chen

**Affiliations:** ^1^ Department of Hepatobiliary Surgery and Fujian Institute of Hepatobiliary Surgery, Fujian Medical University Union Hospital, Fujian Medical University, Fuzhou, China; ^2^ Fujian Medical University Cancer Center, Fujian Medical University, Fuzhou, China; ^3^ Key Laboratory of Ministry of Education for Gastrointestinal Cancer, Fujian Medical University, Fuzhou, China; ^4^ Fujian Key Laboratory of Tumor Microbiology, Department of Medical Microbiology, Fujian Medical University, Fuzhou, China; ^5^ Department of Nursing, Fujian Medical University Union Hospital, Fujian Medical University, Fuzhou, China

**Keywords:** gallbladder cancer, CEP55, DNA damage, apoptosis, proliferation, therapeutic target

## Abstract

**Introduction:**

Gallbladder cancer (GBC) is a highly malignant biliary tumor with a poor prognosis. As existing therapies for advanced metastatic GBC are rarely effective, there is an urgent need to identify more effective targets for treatment.

**Methods:**

Hub genes of GBC were identified by bioinformatics analysis and their expression in GBC was analyzed by tissue validation. The biological role of CEP55 in GBC cell and the underlying mechanism of the anticancer effect of CEP55 knockdown were evaluated via CCK8, colony formation assay, EDU staining, flow cytometry, western blot, immunofluorescence, and an alkaline comet assay.

**Results:**

We screened out five hub genes of GBC, namely PLK1, CEP55, FANCI, NEK2 and PTTG1. CEP55 is not only overexpressed in the GBC but also correlated with advanced TNM stage, differentiation grade and poorer survival. After CEP55 knockdown, the proliferation of GBC cells was inhibited with cell cycle arrest in G2/M phase and DNA damage. There was a marked increase in the apoptosis of GBC cells in the siCEP55 group. Besides, in vivo, CEP55 inhibition attenuated the growth and promoted apoptosis of GBC cells. Mechanically, the tumor suppressor effect of CEP55 knockdown is associated with dysregulation of the AKT and ERK signaling networks.

**Discussion:**

These data not only demonstrate that CEP55 is identified as a potential independent predictor crucial to the diagnosis and prognosis of gallbladder cancer but also reveal the possibility for CEP55 to be used as a promising target in the treatment of GBC.

## 1 Introduction

Gallbladder carcinoma (GBC) is a rare but highly malignant biliary tumor originating from gallbladder mucosal epithelial cells, accounting for 1.2% of all malignant tumors (1). Gallbladder stones and gallbladder polyps with chronic inflammation are important risk factors for GBC. It is one of the few cancers that exhibit a gender bias, with female patients having a three- to six-times higher incidence rate compared to males (2). Meanwhile, GBC is showing increasing morbidity and mortality in some countries and regions, such as China (3). However, GBC cannot be diagnosed until the later stages of the disease or even after metastasis due to the lack of early clinical manifestations. Therefore, patients often miss the best opportunity for surgical treatment, making GBC one of the cancers with an extremely poor prognosis (4). Additionally, there are almost no ideal therapeutic strategies for advanced GBC. With the development of targeted drug therapy, there is hope for the treatment of gallbladder cancer (5–7). Necessarily, it is urgent to find molecular markers and susceptibility genes that can aid in the diagnosis and treatment of gallbladder cancer.

Recently, advanced gene sequencing technology not only provides favorable conditions for tumor research, including gallbladder cancer, but also deepens researchers’ understanding of the precision medicine concept for tumor treatment (8, 9). The pivotal link of precision medicine is to screen therapeutic targets through high-throughput sequencing technology (10), in which bioinformatics analysis is an indispensable step. The screening of tumor-related susceptibility genes based on bioinformatics is helpful to reveal the mechanism of tumorigenesis at the molecular level. Moreover, these screened genes have been identified and successfully used to assist and guide clinical diagnosis and treatment (11). This gives us a hint that it is possible to uncover molecules associated with GBC progression and poor prognosis with the help of bioinformatics analysis. Nevertheless, few studies on gallbladder cancer based on bioinformatics analysis have been reported.

CEP55 (Centrosomal Protein 55), also known as c10orf3 and FLJ10540, is a recently discovered member of the centrosome-associated protein family and plays a key role in cytoplasmic division *via* association with centrosomes and intermediates (12). It has been reported that CEP55 interacts with ESCRT family members and promotes the contraction of intracellular bridges leading to cell division (12). Numerous studies have confirmed that CEP55 influences the performance of midbody-dependent cellular functions, such as centrosome replication (13, 14). Besides, CEP55 shows great potential for enhancing the pluripotent stemness of cancer cells by participating in the autophagic degradation of midbody derivatives (15, 16). The AKT and ERK signaling pathways are demonstrated to exert diverse roles in tumor progression, including apoptosis, angiogenesis, and epithelial-mesenchymal transition (17–20). However, so far, it has remained unknown whether CEP55 is involved in the occurrence and development of GBC, and the role of the AKT and ERK signaling networks in the effect of CEP55 on gallbladder cancer remains to be elucidated.

Here, the hub genes, namely PLK1, CEP55, FANCI, NEK2, and PTTG1, were screened out by analyzing the GBC datasets GSE74048 and GSE76633 in the public database using bioinformatics technology. Subsequently, based on gallbladder cancer tissue validation, we finally determined that CEP55 is a vital gene involved in the progression of gallbladder cancer. Then, by using an *in vitro* CEP55 knockdown system performed by targeted siRNA and an *in vivo* subcutaneous tumorigenesis model in nude mice, we observed that the knockdown of CEP55 markedly restrained the proliferation of gallbladder cancer *via* impeding AKT and ERK pathway activation, resulting inG2/M phase arrest, DNA damage, and apoptosis. Our work not only reveals the previously unrecognized mechanism of CEP55 in the pathogenesis of gallbladder cancer but also shows the possibility for CEP55 to be a promising marker and target to be used in the treatment of GBC and improve the survival rate of patients with GBC, which provides an experimental basis for designing effective targeted therapy drugs for gallbladder cancer.

## 2 Materials and methods

### 2.1 Microarray data

Two Gene Expression Omnibus (GEO) (http://www.ncbi.nlm.nih.gov/geo/) datasets were used in the present study, including GSE74048 (three pairs of human GBC tissues and the matched peri-carcinomatous tissues were included) and GSE76633 (nine pairs of GBC tissues and paired normal gallbladder tissues were included) (21). The database was accessed and data extracted on 23 January 2022. Detailed information on the datasets is presented in **Table S1**.

### 2.2 Identification and enrichment analysis of DEGs

DEGs between GBC tissues and peri-carcinomatous tissues were identified through the GEO2R program (http://www.ncbi.nlm.nih.gov/geo/geo2r) (22). A P-value < 0.05 and a |log(2) fold change (FC)| ≥ 1 was defined as the thresholds for DEG screening. Probe sets without corresponding gene symbols or genes with more than one probe were removed or averaged, respectively. The DEGs that overlapped among the two datasets were analyzed using the “ggplot2 (3.3.3)” package in R (3.6.3). GO and KEGG databases were utilized as references (23), and the “clusterProfiler (3.14.3)” R package carried out an analysis of enrichment.

### 2.3 PPI network construction and module analysis

The protein–protein interaction network analysis among DEGs was conducted using the STRING online database (https://string-db.org/) (24). An interaction with a combined score >0.4 was set as the cut-off value. Then the network was visualized using Cytoscape software (25). The Molecular Complex Detection (MCODE) plugin was used to identify the most significant module in PPI networks with MCODE scores ≥10, degree cut-off = 2, node score cut-off = 0.2, max depth = 100, and k-score = 2.

### 2.4 Hub gene validation and clinical significance

Further GO and KEGG analyses of genes in the top 2 modules were performed, while the DEGs in cluster 1 with the highest score were analyzed using the cytoHubba plugin (26). Specifically, 12 sets of scoring results were obtained using 11 different algorithms. The top 40 genes in various scoring results were selected for intersection by Venn analysis, and the overlapping genes were finally identified as hub genes. The Biological Networks Gene Oncology (BiNGO) plug-in of Cytoscape (27) was applied for the biological process analysis of hub genes. The UCSC Cancer Genomics Browser (28) (https://genome-cancer.ucsc.edu/) was used to construct the hierarchical clustering of hub genes in bile duct cancer samples from the TCGA database. The expression of hub genes in pan-cancer was verified by the GEPIA database (http://gepia.cancer-pku.cn/), and the relationship between hub genes and the survival of patients with pan-cancer was analyzed.

### 2.5 Tissue samples and cell lines

A total of 15 samples of fresh GBC tissues and 174 samples of GBC paraffin specimens and control samples were obtained from patients who were admitted to Fujian Medical University Union Hospital in China and provided written informed consent prior to surgical resection. The samples were collected based on a protocol approved by the Ethics Committee of Fujian Medical University Union Hospital in accordance with the Declaration of Helsinki (application number: 2021QH031). All tumors were confirmed as GBC by the Clinicopathology Department. The human GBC cell lines, NOZ (obtained from the Health Science Research Resources Bank in Japan) and SGC-996 (provided by the Tumor Cytology Research Unit, Medical College, Tongji University, China) were maintained in DMEM (Hyclone, USA) supplemented with 10% FBS (Hyclone, USA). All cells were incubated at 37°C in a humidified atmosphere with 5% CO_2_.

### 2.6 Quantitative real-time PCR analysis

Total RNA was extracted from the GBC tissues or cells with TRIzol reagent (Invitrogen, USA) and reverse transcribed using the All-In-One 5× RT MasterMix (Abm, Canada). PCR was performed with BlasTaq 2× qPCR MasterMix (ABM, Canada), and fluorescence was measured using an Mx3000P QPCR System (Agilent, USA) following the manufacturer’s instructions, with GAPDH as an internal control. The data were analyzed using the −ΔCt or 2^−ΔΔCt^ method. Detailed information on the primer sequences is presented in **Table S2**.

### 2.7 Immunohistochemical analysis and evaluation of CEP55 expression

Immunohistochemistry was performed as previously described (29). Primary antibodies specific for CEP55 (1:400, Abcam) were incubated overnight at 4°C. Signals from samples were developed with DAB reagents (Vector Laboratories, SK-4100) and hematoxylin counterstaining. Images of five random fields per section were obtained under a light microscope (Olympus, Tokyo, Japan). The IHC score was calculated by multiplying the percentage score and intensity score of positively stained cells. The percentage of positively stained cells (0 = 0–10%; 1 = 11–25%; 2 = 26–50%; 3 = 51–75%; 4 = 76–100%) was scored on a scale from 0 to 4. The staining intensity (0 = negative staining; 1 = weak; 2 = moderate; 3 = strong) was scored on a scale from 0 to 3. The IHC score ranged from 0 to 12.

### 2.8 Small interference RNA, short hairpin RNA, and transfection

Two siRNAs targeting the human CEP55 sequence as references (19, 30) were produced by GenePharma (Shanghai, China). Transfection of siRNA was performed with Lipofectamine 3000 (Life Technologies). Short hairpin RNA oligonucleotides targeting CEP55 (shCEP55) and negative control short hairpin RNA oligonucleotides (shNC) were annealed and ligated into the lentiviral vector GV112 (Genechem, Shanghai, China). After 2 weeks, puromycin treatment was used to select for stably infected cells. CEP55 expression in the infected cells was validated by qRT-PCR analysis and Western blot assays.

### 2.9 Cell proliferation assays

Cells were seeded in 96-well plates at a concentration of 2,500 cells/well and incubated with 10% CCK8 reagent (Dojindo Laboratories, Kumamoto, Japan) at 37 °C for 1 h. Cell viability was evaluated using the absorbance values determined at 450 nm by a microplate reader (BioTek, Winooski, VT, USA). Cells were seeded in 96-well plates at 500 cells/well and, after 14 days, fixed in 4% paraformaldehyde for 20 min. Cell colonies were counted after staining with crystal violet (Solarbio, Beijing, China) to assess anchorage-independent growth. DNA replication ability was evaluated by EDU assay (RiboBio, Guangzhou, China). Cells were seeded in 96-well plates at 10^5^ cells/well, labeled with the EDU working fluid for 2 h, washed in PBS, fixed in 4% PFA for 30 min, and stained with Apollo. The images were acquired with Operetta CLS High Content Analysis System (PerkinElmer, USA).

### 2.10 Flow cytometric analysis of cell cycle and apoptosis

Cells were collected and stained with PI/RNase staining buffer (BD Pharmingen™) according to the manufacturer’s instructions to measure cell cycle activity by FACSCanto™ II (BD Biosciences, USA). ModFit LT 5.0 (Verity Software House, USA) was used to analyze the percentage of cell cycle and generate a histogram. Apoptosis was measured with the PE Annexin V Apoptosis Detection Kit I (BD Pharmingen™) according to the manufacturer′s instructions. Cells were collected, washed with cold PBS, gently resuspended in binding buffer containing PE-conjugated annexin-V and 7-AAD, and then incubated at room temperature in the dark for 15 min. The analysis was performed using FACSCanto™ II.

### 2.11 Western blot and antibodies

Cell lysates were extracted using RIPA lysis buffer containing PMSF (Beyotime, Shanghai, China). The protein concentration was measured with a BCA assay kit (Solarbio, Beijing, China). The samples were heated at 95 °C in the loading buffer, separated on SDS-polyacrylamide gels, and transferred to PVDF membranes (Millipore, USA). Then blotted with 5% BSA and incubated with the primary antibody at 4°C overnight. Before incubation with the secondary antibodies, the membranes were washed in TBST. The following primary antibodies were used: Rabbit anti-CEP55 (#81693), anti-γ-H2AX (#9718), anti-p21 (#2947), anti-p53 (#2527), anti-cleaved caspase-3 (#9664), anti-cleaved PARP (#5625), anti-cleaved caspase-9 (#9505), anti-AKT (#9272), anti-p-AKT (Ser473) (#4060), anti-Erk1/2 (#4695), and anti-p-ERK1/2 (Thr202/Tyr204) (#4370) were obtained from CST (Danvers, USA). Rabbit anti-Cyclin B1 (ab181593), anti-Cyclin D1 (ab134175), anti-CDK6 (ab124821), anti-GSK3β (ab32391), and anti-p-GSK3β (Ser9) (ab75814) were purchased from Abcam (Cambridge, UK). Rabbit anti-Bax (A19684), anti-Bcl-2 (A19693), and anti-FoxO3a (A9270) were acquired from Abclonal (Wuhan, China). GAPDH (Servicebio, Wuhan, China) was used as a protein marker for cytosolic proteins. The antibody–antigen complexes were visualized with WesternBrightTM ECL (Advansta, USA).

### 2.12 Alkaline comet assay

The comet assay was performed using the Oxiselect Comet Assay Kit (Cellbiolabs, USA) according to the manufacturer’s instructions under a fluorescence microscope (ZEISS, Germany) with minor modifications. The prepared gel slides containing cells were put into the cell lysate and cracked overnight at 4 °C. Then the gel slides were soaked in the electrophoresis solution for 20 min before electrophoresis. After electrophoresis, the single cells in low melting point agarose were stained in the dark room for 20 min with PI (5 μg/ml). The images were analyzed quantitatively by the software CASP.

### 2.13 Immunofluorescence analysis

After cells were plated on coverslips and cultured overnight, they were washed, fixed, permeabilized, blocked, and stained with anti-γ-H2AX, anti-p-AKT, and anti-p-ERK. Images were observed under a laser scanning confocal microscope (Leica TCS SP5, Germany). Actin-Tracker Red-555, Hoechst 33342, and DAPI staining solution were obtained from the Beyotime Institute of Biotechnology (Shanghai, China).

### 2.14 Subcutaneous xenograft models

BALB/C nude mice (6-week-old, weighing an average of 18–22 g, male) were purchased from Gempharmatech Co., Ltd. (Shanghai, China). All mice were housed in specific pathogen-free conditions following the guidelines of the Ethics Committee of the Medical Faculty of the Fujian Medical University (application number: FJMU IACUC 2021-0435). The nude mice were randomly and equally assigned to two groups and labeled with ear labels. To explore the effects of CEP55 on tumor growth *in vivo*, 1 × 10^5^ NOZ cells (shNC and shCEP55) were suspended in 200 μl serum-free medium and subcutaneously injected into the left axilla of the mice (six mice/group). Tumor growth was monitored every three days and measured in two dimensions. The tumor volume was calculated by the formula: tumor volume = 0.5 × width^2^ × length, where the width and length were the shortest and longest diameters, respectively. The mice were sacrificed after approximately 4 weeks when the maximum tumor volume reached 2,000 mm^3^. Then the tumors were dissected and weighed. Ki-67, cleaved caspase-3, anti-p-AKT, and anti-p-ERK were evaluated in xenograft tumors by immunohistochemical staining (IHC).

### 2.15 TUNEL assay

Cell apoptosis was measured *in vivo* using a TUNEL assay (Roche, Switzerland). After dewaxing, the paraffin sections were repaired with protease K and incubated for 25 min at 37 °C. Then the samples were incubated with 0.1% TritonX-100 at room temperature for 10 min. The samples were incubated in a TUNEL working solution prepared according to the instructions at 37 °C for 2 h. The samples were stained with DAPI for 10 min at room temperature and then observed by a fluorescence microscope (ZEISS, Germany). To calculate the apoptosis rate, the number of positive cells was calculated by randomly selecting three fields under a high-power lens (×400).

### 2.16 Statistical analysis

All data are expressed as the mean ± standard deviation (SD) of independent replicates (n ≥3). SPSS 21.0 software and GraphPad Prism 8.0 software were used for statistical analysis. A t-test statistical analysis was used to compare the mean between the two groups of samples, and a one-way ANOVA statistical analysis was used to compare the mean between multiple groups of samples. The relationship between CEP55 expression in gallbladder carcinoma and adjacent tissues and clinicopathological features was tested by the chi-square test. The difference was statistically significant with a p <0.05.

## 3 Results

### 3.1 Identification, GO enrichment, and KEGG pathway analyses of DEGs

**Figure 1:**
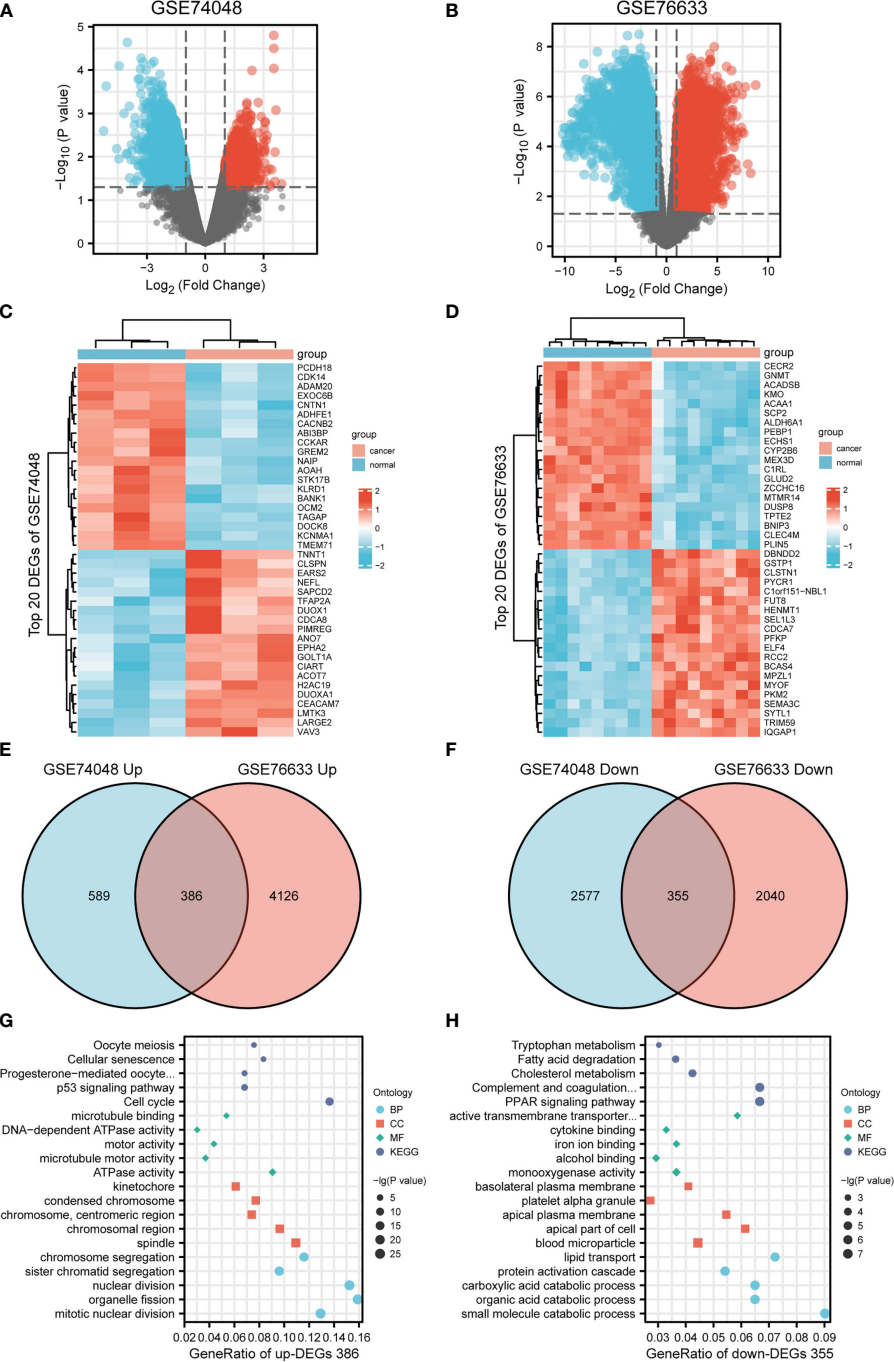
Differentially expressed genes (DEGs) between GBC and paracarcinoma tissues identified in GSE74048 and GSE76633 datasets. **(A, B)** The volcano map visually shows the total DEGs in GSE74048 and GSE76633. **(C, D)** Heat map visualization shows the top 20 DEGs in GSE74048 and GSE76633. **(E, F)** Identification of common upregulated and downregulated genes from DEGs in the GSE74048. **(G, H)** Functional enrichment analysis of common up- and down-regulated DEGs genes in GBC.

To investigate DEGs between GBC tissues and paracarcinoma tissues, we employed the GEO2R online tool to analyze the GSE74048 dataset and the GSE76633 dataset from the GEO database. We identified 3,907 DEGs in the GSE74048 dataset, of which 975 DEGs were elevated and 2,932 DEGs were depressed in GBC tissues compared to paracarcinoma tissues (**Figure 1A** and **Figure S1A**). In the GSE76633 dataset, there were 6,144 DEGs between GBC tissues and paracarcinoma tissues, among which 3,934 DEGs were upregulated and 2,210 DEGs were downregulated (**Figure 1B** and **Figure S1B**). The heatmaps showed the top 20 DEGs of the two datasets (**Figures 1C, D**). To further explore the correlation of DEGs in the two datasets (**Figure S1C**), a Venn diagram was applied and found 386 common upregulated genes and 355 common downregulated genes in GBC tissues as compared to paracarcinoma tissues (**Figures 1E, F**, **Table S3**). In addition, we used the “clusterProfiler” package in R for GO and KEGG pathway enrichment analyses to examine the biological roles of the identified DEGs. The GO and KEGG pathway analysis showed that upregulated DEGs were related to biological processes (including mitotic nuclear division and organelle fission), cellular components (including the spindle and chromosomal region), molecular functions (including ATPase activity and microtubule motor activity), and signaling pathways (connecting the cell cycle and the p53 signaling pathway) (**Figure 1G**). Besides, the enrichment analyses of downregulated DEGs indicated that this group contains genes linked to biological processes (including small molecule catabolic processes and organic acid catabolic processes), cellular components (including the apical part of the cell and the apical plasma membrane), molecular functions (including monooxygenase activity and iron ion binding), and signaling pathways (connecting the PPAR signaling pathway and complement and coagulation cascades) (**Figure 1H**).

### 3.2 Construction of the PPI network and discovery of hub genes

**Figure 2:**
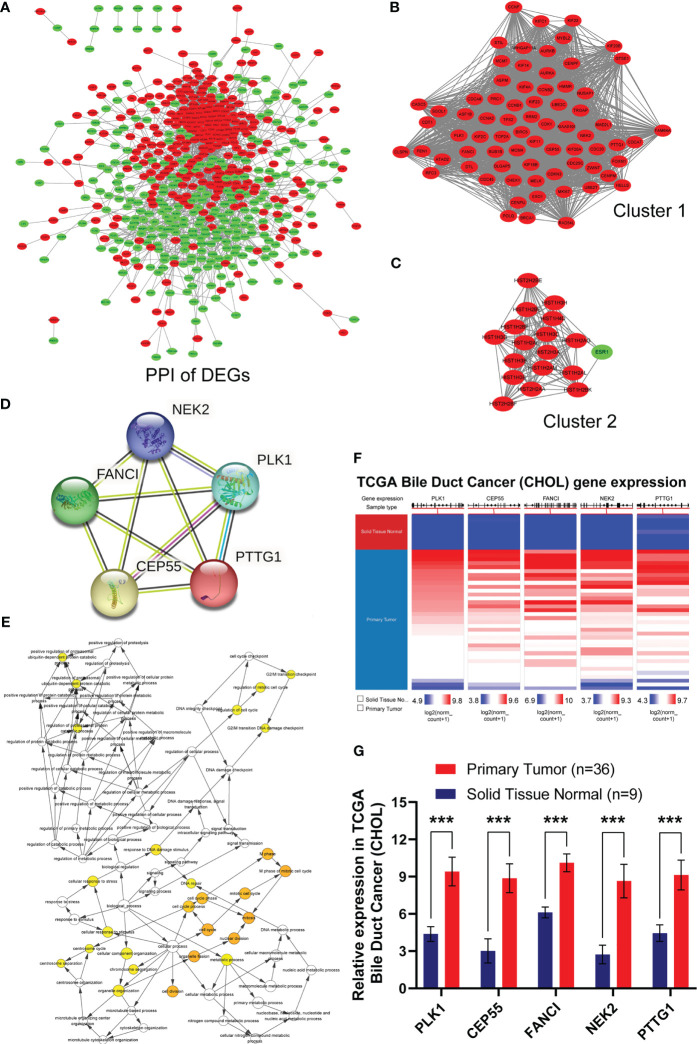
Identification, biological process and differential expression of hub genes from DEGs. **(A)** The PPI network was constructed using common 741 DEGs. Red nodes indicated upregulated genes, and green nodes represented downregulated genes. **(B)** Cluster 1 consisted of 72 nodes and 2317 edges and exhibited the highest score using MCODE plug-in. **(C)** Cluster 2 containing 18 nodes and 134 edges possessed the second highest score. **(D)** PPI network of hub genes in STRING database. **(E)** Biological process of hub genes was analyzed by BiNGO plug-in. **(F, G)** Hierarchical clustering and expression analysis of hub genes in TCGA biliary tract tumors (***P < 0.001).

Based on the data above, we investigated the interaction among the 741 DEGs using a PPI network constructed from STRING with interaction scores >0.4. It was displayed by Cytoscape to elucidate protein interactions, obtaining a total of 614 nodes and 4,911 edges (**Figure 2A**). The MCODE plug-in was used to analyze the PPI network and identify clusters. Cluster 1 consisted of 72 nodes and 2,317 edges, which exhibited the highest score (**Figure 2B**). Furthermore, cluster 2, containing 18 nodes and 134 edges, also possessed strong connections (**Figure 2C**). Based on the GO function and KEGG pathway analyses, these two clusters were principally associated with cell cycle, systemic lupus erythematosus, microtubule binding, nucleosomal DNA binding, spindle, nucleosome, mitotic nuclear division, and nucleosome assembly (**Figures S1D, S1E**). These findings suggested that the mentioned genes showed higher hub degrees and could serve critical roles in GBC.

Subsequently, we employed the cytoHubba plug-in to evaluate the 72 DEGs in cluster 1 with the highest tumor-related closeness through 11 different algorithms and acquired 12 sets of scoring results (**Table S4**). Further Venn intersection analysis of the top 40 genes in various scoring results confirmed the five overlapped genes, namely, PLK1, CEP55, FANCI, NEK2, and PTTG1, as potential hub genes (**Figures S1F–I** and **Figure 2D**). To further clarify the role of the five hub genes, biological process analysis was performed using the BiNGO plug-in. The alterations were mainly enriched in organelle fission, nuclear division, the M phase of the mitotic cell cycle, mitosis, and the cell cycle (**Figure 2E**). Meanwhile, we also observed that the five hub genes were obviously upregulated in bile duct cancer samples (**Figures 2F, G**) and highly expressed in almost all human tumors from TCGA (**Figure S2**) after hierarchical clustering through the UCSC Cancer Genomics Browser. Moreover, pan-cancer patients with upregulated PLK1, CEP55, FANCI, NEK2, and PTTG1 exhibited a poorer overall survival rate and disease-free survival (**Figure S1J**). Taken together, our results demonstrated that the five hub genes have great potential to be markers that distinguish cancer from normal tissues.

### 3.3 CEP55 serves as an underlying promoter and promising predictor of the prognosis in GBC

**Figure 3:**
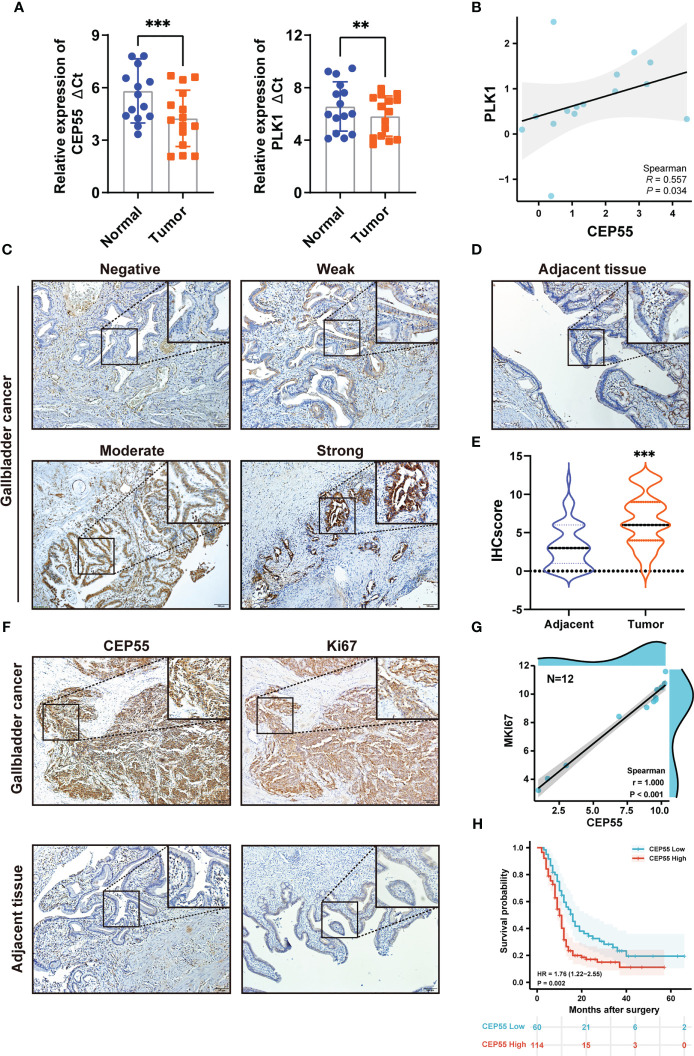
Expression of hub genes in GBC tissues and upregulated expression of CEP55 indicates poor prognosis. **(A)** The mRNA expression levels of PLK1 and CEP55 in GBC tissues. The data are shown as the ΔCt values. **(B)** Analysis of the correlative expression be-tween CEP55 and PLK1. **(C, D)** Protein expressions of CEP55 in GBC and matched paracarcinoma tissues were measured by immunohistochemistry (scale bar, 100 μm). **(E)** Results of immunohistochemical score. **(F)** Analysis of the correlation between CEP55 expression and Ki67 expression in GBC tissues (scale bar, 100 μm). **(G)** Correlation between CEP55 and MKI67 in GSE76633 and GSE74048. **(H)** The relationship between the expression of CEP55 and the overall survival rate (**P < 0.01; ***P < 0.001).

To further determine whether the five hub genes can be used as detectors of GBC, qRT-PCR was used to measure the mRNA expression of these hub genes in 15 human GBC tissue samples. The results showed that the mRNA levels of PLK1 and CEP55 in GBC tissues were significantly higher than those in paracarcinoma tissues, respectively, by 1.67 times (P = 0.006) and 2.97 times (P <0.001) (**Figure 3A** and **Tables S5**, **S6**). While FANCI, NEK2, and PTTG1 showed no statistically significant differences (**Figure S3**). It has been reported that PLK1 is upregulated in gallbladder cancer and is associated with poor prognosis in gallbladder cancer patients (31, 32), while the role of CEP55 in gallbladder cancer has not been reported. In combined analysis, CEP55 was markedly positively correlated with PLK1 in gallbladder cancer, R = 0.557 (**Figure 3B**), confirming that CEP55 may play a vital role in the progression of gallbladder cancer as PLK1. Consistently, immunohistochemistry analysis showed that CEP55 protein was mainly localized in the cytoplasm and increased pronouncedly in GBC tissues compared with adjacent tissues (**Figures 3C–E**). Of note, we found that there was a prominent positive correlation between the expression of CEP55 and Ki67 in immunohistochemical staining of the same batch (**Figure 3F**). Besides, the analysis of two datasets in GEO (GSE76633 and GSE74048) revealed that CEP55 was also positively related to the expression of MKI67 (encoding Ki67 protein, a cell proliferation marker) (R = 1.000, P <0.001), indicating that CEP55 may play a promoter role in the malignant proliferation of gallbladder cancer (**Figure 3G**).

Our above results suggested CEP55’s potential to be used as a marker to help GBC diagnose. Thus, we set out to explore the correlation between CEP55 expression and the clinicopathological characteristics of GBC patients. Notably, it showed that the expression of CEP55 was positively correlated with TNM stage (X^2 ^= 6.345, P = 0.012) and negatively correlated with the degree of differentiation of gallbladder carcinoma (X^2 ^= 6.702, P = 0.010) (**Table 1**). Additionally, Kaplan–Meier curves manifested a sharp reduction of the overall survival rate in patients with elevated CEP55 expression compared with the patients with lower CEP55 (P = 0.002) (**Figure 3H**). Consistently, compared to the low-expression group, the 3-year overall survival rate of the high-expression group was profoundly lower (11.17 vs 23.32%, respectively) (**Table S7**). Meanwhile, the risk ratio of CEP55 overexpression was HR = 1.76 (CI: 1.22–2.55) (**Figure 3H**), revealing that CEP55 may be a promising predictor of the prognosis of patients with gallbladder cancer.

**Table 1:** The relationship between the expression of CEP55 protein and clinical features in gallbladder carcinoma.

Clinicopathological features	Case	Protein expression of CEP55		X^2^	P
		Low (n = 60)	High (n = 114)		
Gender				0.468	0.494
Male	64	20	44		
Female	110	40	70		
Age				1.357	0.244
≤60 years old	54	22	32		
>60 years old	120	38	82		
Gallstone				1.543	0.214
(−)	57	16	41		
(+)	117	44	73		
Differentiation				6.702	0.010
Well or moderate	127	51	76		
Poor	47	9	38		
TNM stage				6.345	0.012
I–II	49	24	25		
III–IV	125	36	89		
Serosa invasion				2.660	0.103
(−)	64	27	37		
(+)	110	33	77		
Lymph node metastasis				0.144	0.705
(−)	101	36	65		
(+)	73	24	49		
Liver invasion				2.759	0.097
(−)	142	53	89		
(+)	32	7	25		

### 3.4 CEP55 augments the growth of GBC cells *in vitro* and *in vivo*

**Figure 4:**
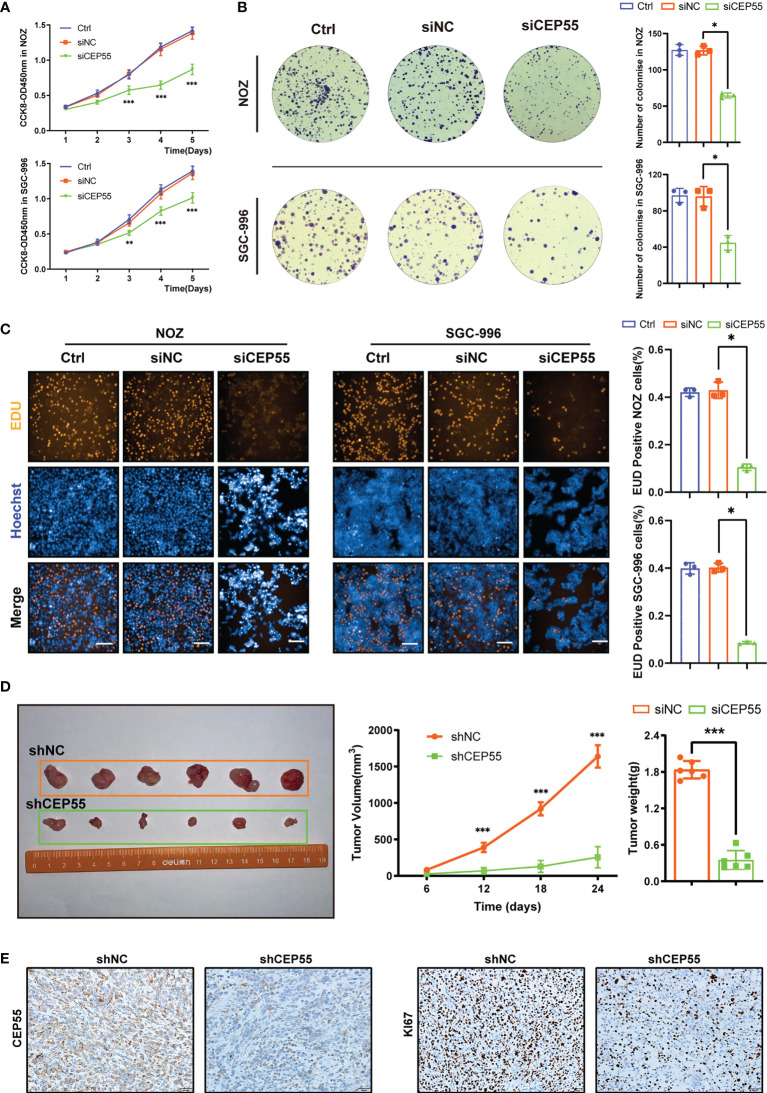
CEP55 knockdown inhibits the growth of gallbladder cancer cells in vitro and in vivo. **(A)** The proliferative activity of cells was detected by CCK8. **(B)** Images and count analysis of colony formation assay. **(C)** Detection of DNA replication by EDU staining and its statistical analysis (scale bar, 50 μm). **(D)** The volume change and weight of gallbladder cancer xenograft tumor. **(E)** Immunohistochemistry to detect the protein expression of CEP55 and Ki67 of gallbladder carcinoma xenograft tumor (scale bar, 50 μm). The experimental group in vitro consisted of three groups, namely, Ctrl (un-treated) group, siNC (negative control) group and siCEP55 (siRNA targeting CEP55) group. The experimental group in vivo consisted of two groups, namely, shNC (negative control) group and shCEP55 (shRNA targeting CEP55) group. The statistical results are based on three and more independent experiments (*P < 0.05; **P < 0.01; ***P < 0.001). NOZ and SCG-996are two cell lines of GBC.

To clarify whether CEP55 promotes GBC cell proliferation, we first transfected NOZ and SGC-996 cells *in vitro* with siRNA to induce the downregulation of CEP55, with siNC and untreated cells used as controls. The reduction efficiency was determined by siRNA knockdown of CEP55, followed by RT-qPCR and Western blotting (**Figures S4A, B**). The CCK-8 assay demonstrated that the proliferative rate of NOZ and SGC-996 cells was impeded by CEP55 knockdown (**Figure 4A**). Consistent with CCK-8 data, the colony formation revealed that the number of colonies in the siCEP55 group was significantly decreased compared with the siNC group (**Figure 4B**). Similarly, as shown, the EDU staining indicated that the proportion of EDU-positive cells was prominently reduced following CEP55 knockdown in NOZ and SGC-996 cells (**Figure 4C**).

In addition, we established tumor xenograft models of GBC in nude mice inoculated with stably transduced cells to investigate the effect of CEP55 on gallbladder cancer growth *in vivo*. A lentiviral vector expressing the siCEP55 construct (shCEP55) as well as a control vector (shNC) were used to establish cells that stably expressed shRNA for CEP55 knockdown or a control shRNA. The protein levels of CEP55 in the shCEP55 cells were sharply decreased relative to those in the wild-type and shNC cells (**Figure S4C**). As expected, there was a striking decline in the xenograft tumor volume and weight in the shCEP55 group compared with the shNC group (**Figure 4D**). Besides, immunohistochemical staining showed that the expression of Ki67 was decreased in the shCEP55 group as compared with the shNC group, which was consistent with the results of the *in vitro* experiment and bioinformatics analysis (**Figure 4E**). Collectively, these results demonstrated the notion that CEP55 augments the proliferation of GBC cells *in vitro* and promotes the growth of GBC in tumor xenograft models of GBC.

### 3.5 Cell cycle arrest at G2/M checkpoint occurs in GBC cells of CEP55 knockdown

**Figure 5:**
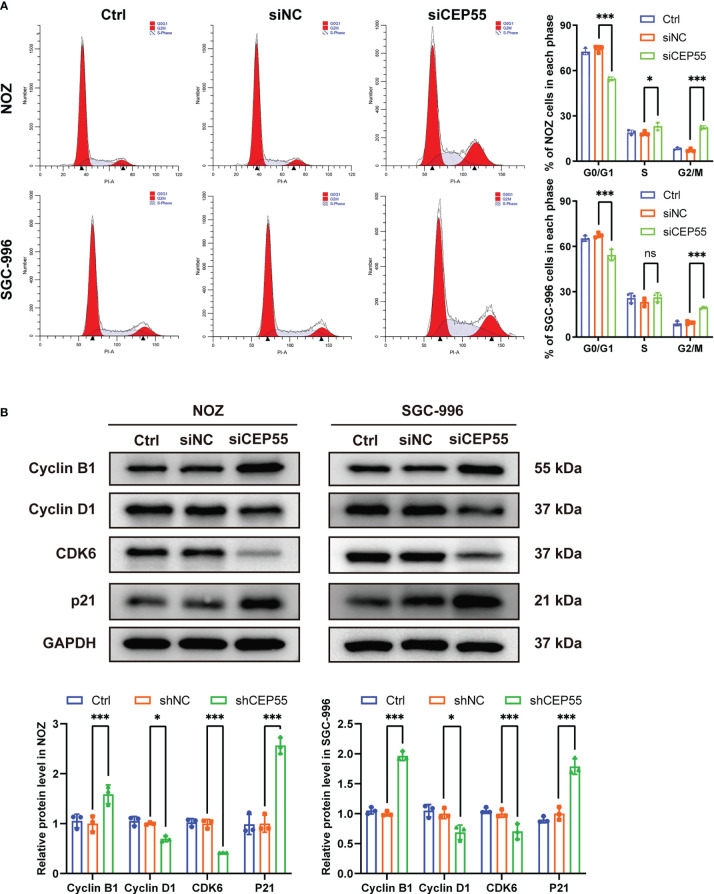
CEP55 knockdown induces gallbladder cancer cells cycle arrest in G2/M phase. **(A)** The cell cycle was detected by flow cytometry. **(B)** Detection of cell cycle related protein expression by western blot. The statistical results are based on three independent experiments (*P < 0.05; ***P < 0.001).

To further reveal the mechanism behind CEP55 affecting the growth of gallbladder cancer cells, flow cytometry was employed to test the changes in cell cycle after CEP55 knockdown in NOZ and SGC-996 cells. The results showed that the proportion of cells in G0/G1 phase was significantly reduced in the CEP55 knockdown group compared with the control group. In contrast, the proportion of cells in G2/M phase was significantly increased, while the proportion of S phase cells did not change much (**Figure 5A**). Western blot was used to assess the expression of cell cycle-related proteins in NOZ and SGC-996 cells. Consistently, we found that the expression of CyclinD1 and CDK6 was significantly downregulated, but the expression of CyclinB1 and P21 was sharply upregulated in the CEP55 knockdown group (**Figure 5B**), which suggested that G1 phase regulatory proteins were inhibited and G2/M phase regulatory proteins were activated after CEP55 knockdown. Based on the data above, we speculate that cell cycle arrest at the G2/M checkpoint occurs in the GBC of CEP55 knockdown, which results in impeding cell proliferation mediated by mitotic failure.

### 3.6 CEP55 knockdown promotes DNA damage even apoptosis in GBC cells

**Figure 6:**
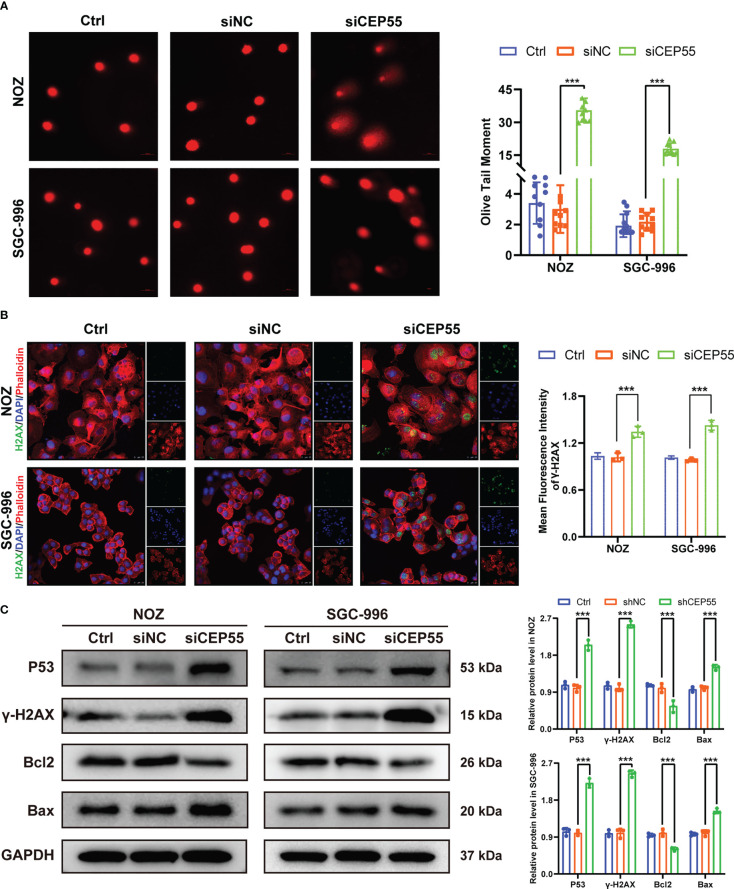
CEP55 knockdown induces DNA damage gallbladder cancer cells. **(A)** Alkaline comet assay to detect DNA damage in gallbladder cancer cells (scale bar, 50 μm). **(B)** Immunofluorescence detection of γ-H2AX expression in gallbladder cancer cells (scale bar, 25 μm). **(C)** Detection of DNA damage-related protein expression in gallbladder cancer cells by western blot. The statistical results are based on three independent experiments (***P < 0.001).

**Figure 7:**
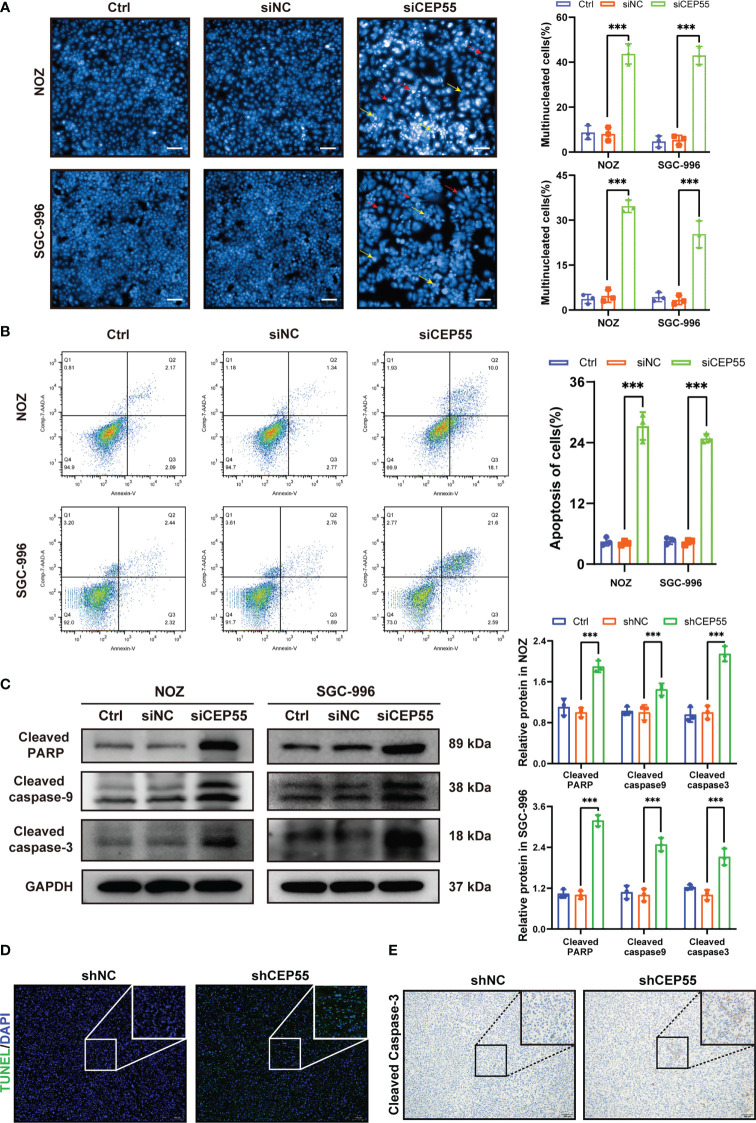
CEP55 knockdown promotes apoptosis of gallbladder cancer cells. **(A)** Hoechst33258 staining to observe the morphology and statistical analysis of gallbladder cancer cell nuclei (scale bar, 50 μm). The red arrows represent binuclear or even multinucleated cells, and the yellow arrows represent nuclear fragmentation and nuclear condensation. **(B)** Detection of apoptosis in gallbladder cancer cells by flow cytometry. **(C)** The ex-pression of apoptosis protein in gallbladder cancer cells was detected by western blot. **(D)** The apoptosis of gallbladder cancer cells was detected by TUNEL assay (scale bar, 50 μm). **(E)** The expression of cleaved caspase-3 protein in xenograft tumors were de-tected by immunohistochemistry (scale bar, 50 μm). The statistical results are based on three and more independent experiments (***P < 0.001).

Growing evidence indicates that severe cell cycle arrest induces DNA damage (33, 34). To further investigate whether the cell cycle arrest after CEP55 knockdown led to DNA damage in NOZ and SGC-996 cells, an alkaline comet assay was performed to evaluate the change in DNA damage. There was an obvious trailing phenomenon in the cells of the silenced group compared with the control group, suggesting that the CEP55 knockdown cells underwent DNA damage (**Figure 6A**). Meanwhile, immunofluorescence showed that the staining intensity of γ-H2AX was significantly enhanced in the silenced group of cells (**Figure 6B**). Furthermore, Western blot results were consistent with the immunofluorescence results (**Figure 6C**). Compared with the control group, the expression of γ-H2AX, p53, and Bax in the siCEP55 group was increased, but the expression of Bcl-2 was decreased (**Figure 6B**). It was indicated that severe cell cycle arrest in the GBC cells of CEP55 knockdown leads to DNA damage. Previous studies have revealed that cell cycle arrest and DNA damage can promote cell apoptosis (35, 36). We employed Hoechst33258 staining to determine the effects of CEP55 knockdown on nuclear morphological changes in GBC cells. The results showed that after knocking down CEP55, NOZ, and SGC-996 cells, they appeared binuclear or even multinuclear, and their nuclear morphology was fragmented and condensed. Quantitative analysis showed that the proportion of multinucleation and nuclear fragmentation in gallbladder cancer cells increased after knocking down CEP55 (**Figure 7A**). Consistently, flow cytometry with Annexin-V-PE and PI staining exhibited that the proportion of cells with early apoptosis (Q3) and late apoptosis (Q2) was significantly higher in the siCEP55 group, but there was no difference between the Ctrl group and the siNC group (**Figure 7B**). Additionally, the apoptosis-related proteins Cleaved-PARP, Cleaved caspase9 and Cleaved caspase3 were upregulated in the silenced group (**Figure 7C**). Furthermore, the TUNEL assay and immunohistochemistry and applied to verify the effect of CEP55 on the apoptosis of GBC xenografts. In the TUNEL assay, there were more TUNEL-positive cells were observed in the CEP55 knockdown tumor (**Figure 7D**). Furthermore, the results of cleaved caspase-3 immunohistochemistry were consistent with those of TUNEL (**Figure 7E**). Overall, it was suggested that apoptosis occurs after silencing the expression of CEP55 in gallbladder cancer cells.

### 3.7 The tumor suppressor effect of CEP55 knockdown is associated with dysregulation of AKT and ERK signaling pathways

**Figure 8:**
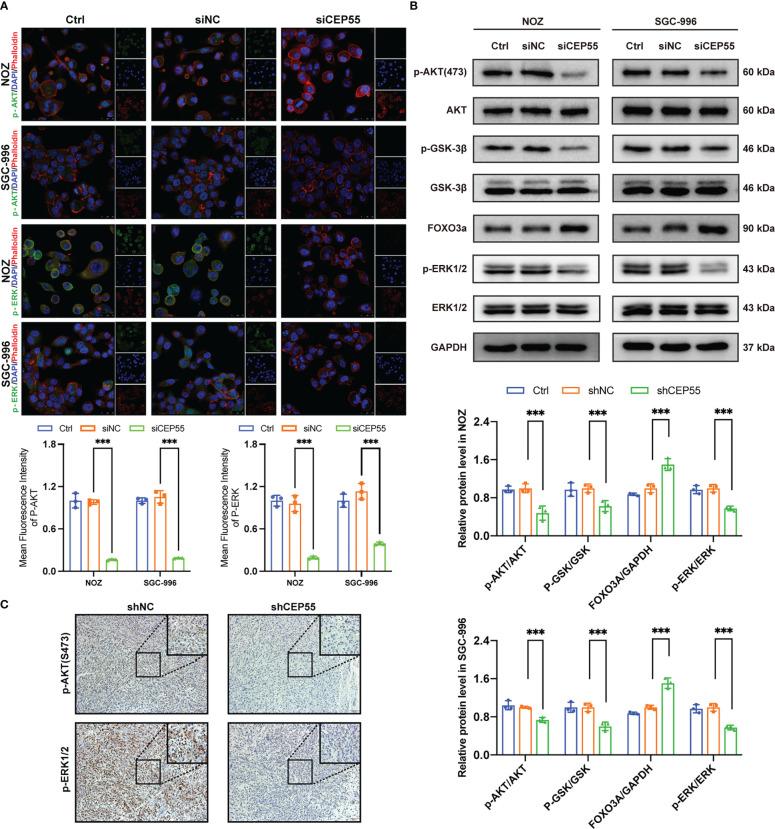
The tumor suppressor effect of CEP55 knockdown is associated with dysregulation of AKT and ERK signaling pathways. **(A)** Immunofluorescence detection of p-AKT and p-ERK expression in gallbladder cancer cells (scale bar, 25 μm). **(B)** Detection of AKT and ERK pathways related protein expression by western blot. The statistical results are based on three independent experiments (***P < 0.001). **(C)** The expression of p-AKT and p-ERK1/2 protein in xenograft tumors were detected by immunohistochemistry (scale bar, 50 μm).

Studies have shown that CEP55 upregulates the phosphorylation of AKT by directly combining with the subunit P110 of PI3K, thus promoting cell proliferation *in vitro* (37, 38). To further decipher the molecular signaling network involved in CEP55 accelerating the proliferation and survival of gallbladder cancer cells, we employed immunofluorescence assays to detect the effect of downregulation of CEP55 on AKT and ERK-dependent signaling pathways. The results showed that the fluorescence intensity of p-AKT and p-ERK1/2 expression in gallbladder cancer cells decreased significantly after CEP55 knockdown (**Figure 8A**). In addition, the results of western blot showed that the phosphorylation levels of AKT (Ser473), GSK-3 β (Ser9), and ERK1/2 (Thr202/Tyr204) decreased in gallbladder cancer cells after CEP55 knockdown, but the protein expression level of FOXO3a increased in the siCEP55 group (**Figure 8B**). Furthermore, the immunohistochemical results of transplanted tumor sections in nude mice were consistent with the above results, showing that the expression of p-AKT and p-ERK1/2 decreased significantly in the shCEP55 group compared with the shNC group (**Figure 8C**). The above results suggest that the inhibition of gallbladder cancer cell growth after knocking down CEP55 is related to the cascade inactivation of the AKT and ERK signaling networks.

## 4 Discussion

Due to our limited understanding of gallbladder cancer etiology and pathogenesis, there is a huge obstacle to its prevention and treatment. It is of great significance to discover susceptible genes related to GBC and further reveal its molecular pathogenesis. Excitingly, the application of bioinformatics analysis presented a new insight into exploring molecularly targeted drugs for gallbladder cancer. In our study, it was found that the GO analysis revealed that upregulated DEGs were mainly participated in cell division and cell cycle regulation, while the downregulated DEGs were related to small molecule catabolism and organic acid catabolism. In addition, it displayed cell cycle and P53 signaling pathways obtained by the KEGG analysis of upregulated DEGs, and the downregulated DEGs were mainly enriched in the complement pathway and PPAR signaling pathways. Notably, we noticed that five screened hub genes, including PLK1, CEP55, FANCI, NEK2, and PTTG1, are also closely associated with cell division and proliferation. Meanwhile, a database search found that these hub genes were highly expressed in TCGA bile duct cancer tissue. Therefore, it is speculated that these five hub genes may be important markers for the diagnosis and therapy of gallbladder cancer. It was worth noting that the mRNA levels of PLK1 and CEP55 were significantly upregulated in gallbladder cancer tissues than in adjacent tissues. However, there was no significant difference in the expression of FANCI, NEK2, or PTTG1, which may be due to the insufficient sample size. Strikingly, previous studies demonstrated that PLK1 is highly expressed in GBC tissues, advances GBC progression, and exacerbates the prognosis of gallbladder cancer (31, 32). But the expression of CEP55 in gallbladder cancer and its role in the progression of gallbladder cancer remains to be elucidated. Correlation studies suggested that the expression of CEP55 in gallbladder cancer was significantly positively correlated with that of PLK1, indicating that CEP55 has potential as a molecular target for gallbladder cancer therapy like PLK1. Accumulating evidence indicates that CEP55 expression was elevated in some other tumors, such as liver cancer (38), cervical cancer (39), and lung cancer (40). Likewise, the protein expressions of CEP55 were obviously increased in 174 gallbladder cancer tissues. Moreover, we observed that CEP55 overexpression in gallbladder cancer was positively correlated with TNM stage and negatively correlated with the degree of differentiation of gallbladder cancer, which implies that the detection of CEP55 in gallbladder cancer tissues may help to assess the progressive status of gallbladder cancer patients more accurately. Expectedly, subsequent Kaplan–Meier analysis and Cox regression analysis suggested that CEP55 was suitable as an independent predictor to help determine the prognosis of patients with gallbladder cancer.

CEP55 was identified as a coiled-coil centrosome protein that plays a critical role in many physiological and pathological processes. Furthermore, a few studies have shown that CEP55 is involved in the proliferation process of kidney cancer cells (41), NSCLC cells (42), and neuroblastoma cells (43). Consistent with previous studies, we found that the proliferation ability of gallbladder cancer cell lines NOZ and SGC-996 was obviously reversed after CEP55 knockdown. Moreover, the subcutaneously transplanted tumor in nude mice in the CEP55 silent group was significantly smaller than that in the control group. Besides, immunohistochemical staining showed that Ki-67, a marker representing cell proliferation, was downregulated in the silent group. Overall, we demonstrated that high expression of CEP55 promotes the proliferation of gallbladder cancer cells *in vitro* and *in vivo*. An abnormal cell cycle is one of the fundamental mechanisms of tumorigenesis. To date, growing evidence indicates that overexpression of Aurora A/B, cyclin D/E, Cdc20, Skp2, and PLK1 has been frequently detected in all kinds of cancers, and these oncoproteins serve as effective therapeutic targets in the clinic (44). In our study, it is noteworthy that the mitosis failure of GBC cells leads to cell cycle arrest after knocking down CEP55, which triggers the inhibition of G1 phase regulatory proteins like CyclinD1 and CDK6, and the activation of G2/M phase regulatory proteins like CyclinB1. Furthermore, it is noteworthy that the cycle inhibitory protein P21 was also elevated.

Disruption of the cell cycle results in polynucleated polyploid cells that are prone to genomic instability and induced DNA damage, which is one of the reasons why failure of mitosis activates cell death (45, 46). CEP55 is an important regulator involved in cytoplasmic division, and its abnormal expression is clearly associated with genomic instability (47, 48), which is an important sign of malignant tumors (49). Here, we observed that gallbladder cancer cells after CEP55 knockout showed obvious tailing and appeared binucleate or even multinucleate, indicating the instability of the gallbladder cancer cell genome and DNA damage. The level of γ-H2AX effectively reflects the degree of DNA damage and repair (50, 51). Expectedly, the expression of γ-H2AX was significantly increased in the CEP55 silent group. Therefore, gallbladder cancer cells show DNA damage because of cell division failure after knocking down CEP55. Recently, it has been shown that DNA damage is an important factor in the induction of apoptosis (52). Additionally, the tumor suppressor protein p53 prevents cells with damaged DNA from dividing and inhibits tumor formation (53, 54). In the event of severe DNA damage that is difficult to repair, p53 induces apoptosis (55, 56). Notably, after knockdown of CEP55, the expression of p53 and Bax in GBC cells was dramatically upregulated while the expression of Bcl-2 declined. It has been reported that p53 can upregulate the expression of p21, which leads to cell cycle arrest by binding to Cyclin E/CDK2 and Cyclin D/CDK4 (57). This provides a reasonable explanation for the simultaneous increase of P53 and P21 in gallbladder cancer cells after knockdown of CEP55 in this study. Moreover, it was found that the nuclei of GBCs were fragmented and condensed, and the expression of apoptosis proteins in GBCs was also obviously raised after CEP55 knockdown. Consistently, it showed an elevated apoptosis rate. In addition, the fluorescence intensity of TUNEL staining and the positive ratio of cleaved caspase-3 in the silent group were also increased. Combined with the above results, it suggested that the severe DNA damage that occurred after CEP55 knockdown induced apoptosis. Apoptosis has been shown to have a sophisticated relationship with the abnormal proliferation of tumor cells (58). Apoptosis and cell cycle alterations promoted by DNA damage have been demonstrated to regulate cancer cell proliferation in colorectal cancer (59), liver cancer (60), and lung cancer (61). Overall, the failure of mitosis in gallbladder cancer cells after knockdown of CEP55 causes severe DNA damage and even apoptosis, which in turn inhibits cell proliferation.

Increasing evidence suggests that activation of the AKT and ERK pathways plays an important role in CEP55’s promotion of tumor progression. It has been reported that the interaction between CEP55 and SPAG5 triggered the phosphorylation of AKT at Ser473 and activated the PI3K/AKT pathway to exhibit pro-hepatocellular carcinoma activities (18). Meanwhile, Liu suggested that miR-34a/c induced the apoptosis of caprine endometrial epithelial cells by binding CEP55 *via* the ERK and AKT pathways (17). Additionally, the researchers implied that CEP55 positively influenced the tumorigenesis of esophageal squamous cell carcinoma by enhancing the colony formation and migration of ESCC cells through the phosphorylation of Src, FAK, and ERK (40). Consistent with previous studies, the results in our study demonstrated that the knockdown of CEP55 downregulated the phosphorylation level of AKT, GSK-3β, and ERK1/2 to inhibit AKT and ERK signaling pathways in gallbladder cancer cells, causing the cascade inactivation of the AKT and ERK signaling networks.

Collectively, our studies led us to conclude that CEP55, highly expressed in gallbladder carcinoma, can be used as a potential independent predictor for disease diagnosis and prognostic prediction. Moreover, knockdown of CEP55 in gallbladder cancer cells ultimately affects the proliferative activity of the cells through AKT and ERK pathways mediated by cell cycle arrest, DNA damage, and even apoptosis. Our findings provide further insight into why CEP55 is a target for precision medicine therapy for gallbladder cancer.

## Data availability statement

Publicly available datasets were analyzed in this study. This data can be found here: GSE74048 and GSE76633 from GEO database.

## Ethics statement

The studies involving human participants were reviewed and approved by Ethics Committee of Fujian Medical University Union Hospital. The patients/participants provided their written informed consent to participate in this study. The animal study was reviewed and approved by Ethics Committee of the Medical Faculty of the Fujian Medical University. Written informed consent was obtained from the individual(s) for the publication of any potentially identifiable images or data included in this article.

## Author contributions

Conceptualization, YC, FS, and MH. Methodology, FZ, MC, and LH. Validation, MH, FZ, and WC. Formal analysis, FZ, WC, and LH. Investigation, MH and XA. Data curation, FZ and MC. Writing—original draft preparation, MH. Writing—review and editing, FS. Visualization, MH and FZ. Resources, supervision, and project administration, FS and YC. Funding acquisition, YC. All authors listed have made a substantial, direct, and intellectual contribution to the work and approved it for publication.
