# Supplementary material for: CEP55 as a promising biomarker and therapeutic target on gallbladder cancer
Source: Front Oncol. 2023 May 18;13:1156177. doi: 10.3389/fonc.2023.1156177 (PMC10232967; doi:10.3389/fonc.2023.1156177)
Supplement: Supplementary file 1 [file DataSheet_1.pdf]

Supplementary Material

CEP55 as a promising biomarker and therapeutic target on gallbladder cancer

Maotuan Huang[1,2,3,4,†], Fuxiu Zhong[1,2,5,†],Mingyuan Chen[1,2,3,4], Lingju Hong[1,2,3,4], Weihong Chen[1,2,3,4], Xiahenazi Abudukeremu[1,2,3,4], Feifei She[3,4,*], Yanling Chen[1,2,3,4,*]

[1]Department of Hepatobiliary Surgery and Fujian Institute of Hepatobiliary Surgery, Fujian Medical University Union Hospital, Fujian Medical University, Fuzhou, China.

[2]Fujian Medical University Cancer Center, Fujian Medical University, Fuzhou, China.

[3]Key Laboratory of Ministry of Education for Gastrointestinal Cancer, Fujian Medical University, Fuzhou, China.

[4]Fujian Key Laboratory of Tumor Microbiology, Department of Medical Microbiology, Fujian Medical University, Fuzhou, China.

[5]Department of Nursing, Fujian Medical University Union Hospital, Fujian Medical University, Fuzhou, China.

†These authors contributed equally to this work

*** Correspondence:** Y. Chen, chenyanling@fjmu.edu.cn; F. She, shefeifei@yeah.net.

1 Supplementary Figures and Tables

1.1 Supplementary Figures

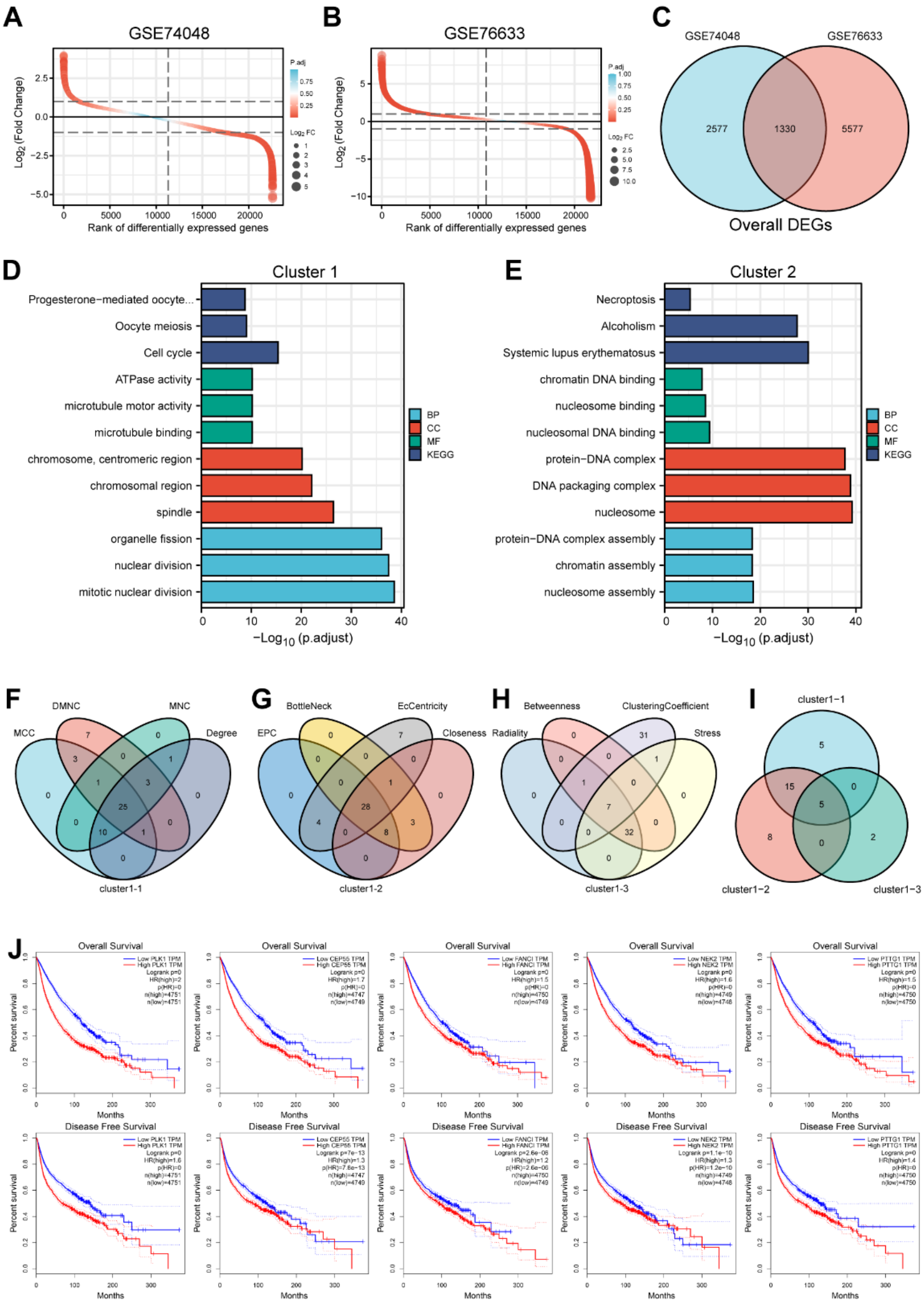

Supplementary Figure 1. DEGs identified in GSE74048 and GSE76633 datasets and analysis of hub genes. (A-B) Sequence Diagram of DEGs in GSE74048 and GSE76633. (C) The common DEGs of GSE74048 and GSE76633. (D-E) GO enrichment and KEGG pathways analysis of DEGs from top 2 clusters. (F-I) The DEGs in cluster 1 were analyzed by CytoHubba plug-in, and the score results were

collected and intersected to determine the core genes. (J) Relationship between hub genes and survival rate in patients with pan-cancer.

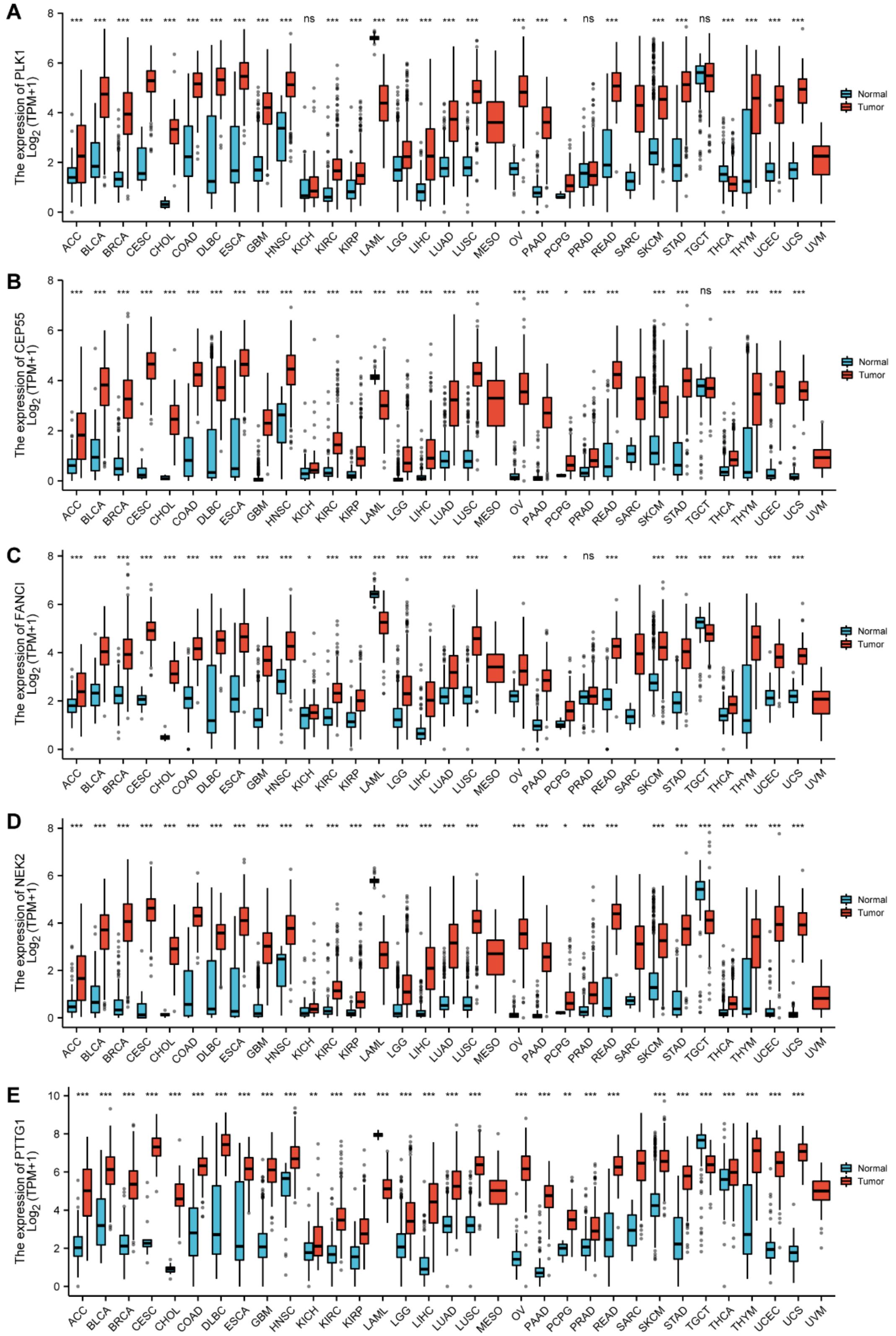

Supplementary Figure 2. The 5 hub genes were highly expressed in almost all human tumors. The expression of (A) PLK1, (B) CEP55, (C) FANCI, (D) NEK2 and (E) PTTG1 in pan-cancer (*P < 0.05; **P < 0.01; ***P < 0.001).

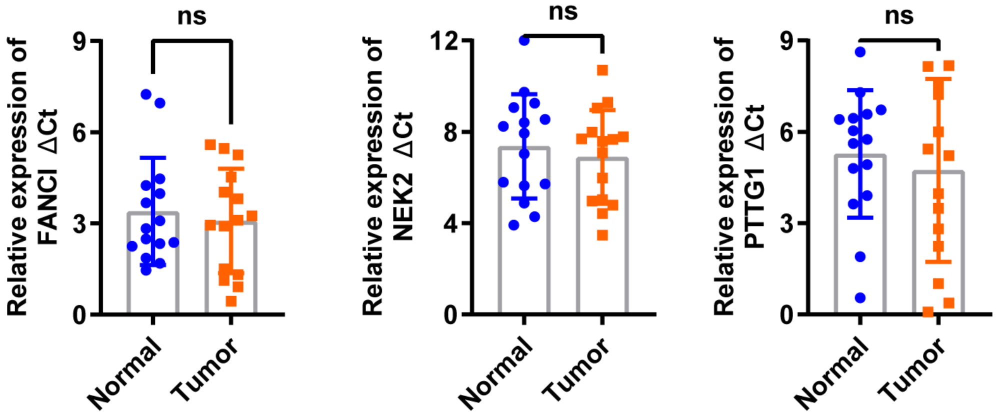

Supplementary Figure 3. The mRNA expression levels of FANCI, NEK2 and PTTG1 in GBC tissues. The data are shown as the ΔCt values.

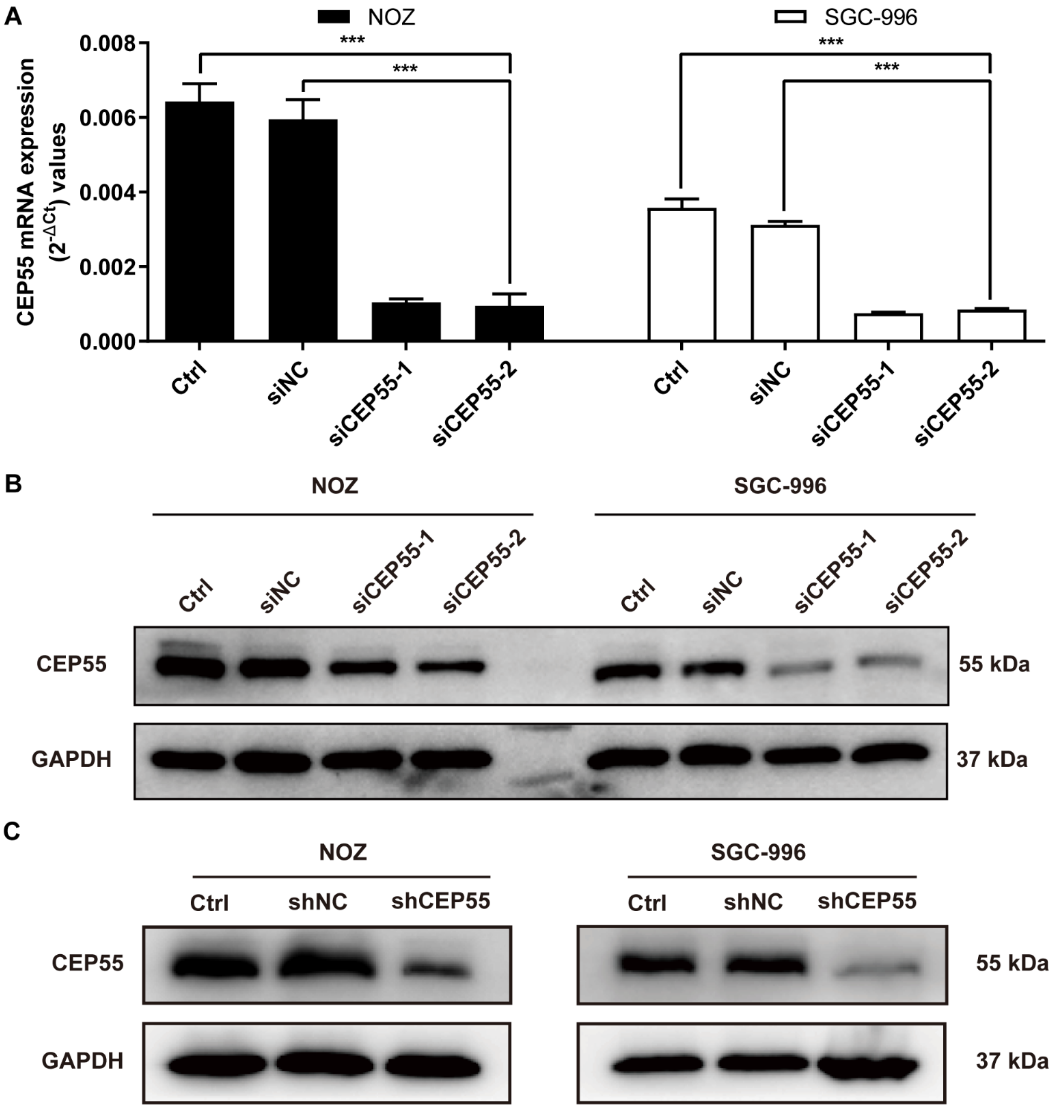

Supplementary Figure 4. The expression of CEP55 in GBC cells was knocked down by RNAi technique. (A-B) The expression of CEP55 in NOZ and SGC-996 cells was inhibited by siRNA targeted to CEP55. (C) Western blot to analyze the expression of CEP55 protein in NOZ and SGC-996 cells stably transduced with shCEP55 or shNC. The statistical results are based on three repeated experiments (***P < 0.001).

1.2 Supplementary Tables

Supplementary Table 1. Detailed information of the GEO datasets in this study.

Series accession	Species	Type	Platform

GSE76633	Homo sapiens	Expression profiling by array	GPL18180 Agilent -045142 Human LncRNA v44X180K
GSE74048	Homo sapiens	Expression profiling by array	GPL20115 Agilent-067406 Human CBC lncRNA + mRNA microarray V4.0

Supplementary Table 2. Detailed information of the primer sequences in this study.

Gene	Primer Sequence
CEP55	F 5’-TGCACACTTAAAGACATCAGTGG-3’
	R 5’- CAGTTGGTCTCTCAGTCGCT-3’
PLK1	F 5’- GCAAGTGGGTGGACTATTCG-3’
	R 5’- TGTAGAGGATGAGGCGTGTT-3’
FANCI	F 5’- CCAGCAAGCTGATGTTCGAC-3’
	R 5’- TCAGAGGAGGCAGCAGATCA-3’
NEK2	F 5’- GGATTACCATCGACCTTCTGTTG-3’
	R 5’- GCTCTCGCTCCTGTAACTGA-3’
PTTG1	F 5’- TCTGGACCTTCAATCAAAGCCT-3’
	R 5’- GTTTGAGGGGTCCCTTGGTC-3’
GAPDH	F 5’- AGGGCTGCTTTTAACTCTGGT-3’
	R 5’- TCTCGCTCCTGGAAGATGGTG-3’

Supplementary Table 3. A total of 741 DEGs were identified from two profile datasets, including 386 upregulated and 355 downregulated genes in GBC compared with paracarcinoma tissues.

DEGs	Gene names
Up-regulated	TRAF2 ESCO2 AURKB EML2 CNKSR1 CDCA7L KRT19 ENO1 LAMB3 IQGAP3 MST1R TIPIN KAZALD1 HSPB1 PFKP DIAPH3 ZNF587 NVL IRAK1 MKI67 EPS8L1 SGPP2 ARHGAP11A MTHFD2 CXCL3 AP1S3 SOX9 PRSS22 LMO7 GIT1 IER3 FRAS1 CLSPN DTL KLK6 HIST2H2AA4 TPX2 EPPK1 AGFG1 RAB27B TM4SF1 SLC29A2 GPSM2 JUP MUC12 OSBP2 CENPQ SIRT7 MRTO4 DDX11 IGF2BP3 FANCA INF2 CCNB1 STC1 SGOL1 ERVMER34-1 TSPAN15 LMTK3 CDRT1 KIF20B FOXQ1 ITGB1BP1 CD2BP2 VSIG1 HIST1H3H C6orf132 TMEM51 IGF2BP2 ASPM SLC12A8 KRT80 IGSF3 HMMR KIF4A LAMC2 ANKRD22 HSPA1B KCTD13 PSMG3 CXCL5 ENTPD6 FAT1 B3GNTL1 RFC3 ARL4A STX1A GPATCH4 CHAF1B CCDC34 DUOXA1 FAM83B ITGA3 BRCA1 SAMD1 EXO1 SLC35F2 HIST1H3E PHF19 CLDN4 ARL14 PKDCC ETV7 MSH5 ETV4 PIGZ KIAA0907 HIST2H3A ANXA3 RASSF10 CD2AP ATAD2 CENPI MED25 PYGB CCNF CLIC3 PPP1R14B UNC93B1 HIST1H2AM RIPK3 TMCO3 NUAK2 EIF5A2 PAX6 KIF2C C15orf52 BIRC5 KIF20A WDR54 SEZ6L2 WHSC1 PLK1 IQCE BCAR1 MRPL52 ZNF28 IER5L KLK13 TNFRSF6B STIL ANKFN1 ELF4 UBE2C MUC13 KLK8 TOMM40 TRIM46 GTF2IRD1 CHML BRMS1 FOXM1 GSG2 MYO19 PYCR1 CCNB2 HOXC9 CHTF18 HIST2H2BF MCM7 HIST1H3G APEX2 PRC1 HIST1H4D FOXD4 TRIM31 TINAG GRIN2D HIST1H2AD CASC5 PKP3 FRMD5 CDK1 PRTFDC1 HIST1H3B CDCA7 PPDPF CDT1 CCDC137 KIF22 CHEK1 GPRIN1 MELK SFN LRP8 DCTPP1 CENPW TONSL KIF11 ALDH3B1 OVOL2 BEND3 CEP55 NDOR1 CDK16 HTATIP2 SPDEF EFNA4 AATF HES4 MLF1 CDC20 PRAME ANKRD18A PPP1R9A TNFSF15 CDR2L KIF18B RAB25 RRM2 HN1 TRIM16L ZWINT MUC17 MRPL17 TUBB3 KIF9 ASF1B CDKN2A EN1 TOP2A SLC4A3 KIAA0101 FEN1 TFRC ENOPH1 PODXL2 TNNI3 FGF18 REC8 HELLS ILF3 FANCI SPTSSB MUC4 H2AFX ADAM15 CCNA2 HIST1H2AG S100A16 C3orf52 TUBG1 SLC16A3 MYO5C BACE2 LIF RAD54L FANK1 PLAGL2 CDC25C GTSE1 CDC45 TMEM164 SLC9A1 STIP1 PLEC CDKN3 NME1 POLQ CLCF1 SPINT2 PMAIP1 LAMA3 ASNS HIST1H2BE ALDOA HIST1H2AI RCC1 TBX18 LIG1 FAM160A1 C21orf58 HIST1H3C WDR62 VAV3 HIST1H2AL TCIRG1 MSTO1 CTTN INPP5J KIFC1 MACC1 SLC1A5 ATG9B ULBP3 LAPTM4B RPL27A MCM4 CA12 C19orf45 MYBL2 GULP1 PTTG1 BCL9L SLC25A19 TGFA ARHGAP11B NEK2 TROAP HIST2H2BE TRIM28 CENPM ATP2C1 BOLA2B DDX50 RASEF CNIH4 RBL1 CENPO CEACAM7 AURKA BZW2 GGCT UBE2T MNX1 ARHGEF38 FAM64A SPC24 CENPF OTX1 RABEP2 NUSAP1 HIST1H2BK MSH2 EPCAM TRIM47 MVP KIF14 KCNJ14 CANT1 LIN9 TFAP2A C2CD4A BUB1B PPP1R37 C17orf53 LYPD1 CUZD1 MAD2L1 DKK1 BCAS4 XPO5 TNNT1 DUOX1 DLGAP5 L3MBTL1 BIRC3 ABHD11 FAM60A KIF23 PGK1 EEF1A2 E2F2 PAQR4 CDCA8 HIST1H2BF XPR1 XLOC_002867 C9orf140 KIAA1244 CTSL2 C9orf100 XLOC_l2_007884 C9orf167 C17orf28 XLOC_l2_009441 LOC100130009 LOC283028 AZI1 ANKRD18B LEPREL4 HIST1H2AM C15orf23 C6orf26 XLOC_010390

	LOC100653149 C19orf21 C15orf42 MLF1IP FAM190A C4orf21 FAM100B C1orf135 LOC81691 C17orf76 RDBP
Down-regulated	RBP5 CHPT1 SCN7A PRKCE CMBL DEFA3 ACSS3 PEG3 TUBE1 CFHR2 GREM2 PCDH9 MPDZ SRCIN1 ABCD3 ZNF554 RIC3 MTUS2 ABCA8 FBLN7 TAS2R60 SOCS2 SLCO1B7 SEC14L3 ACOT12 AIFM1 TSLP CFHR5 IGF1 MPPED2 VWA5B1 DPT TDRD10 C1S ANXA6 SLC22A11 CDADC1 GNA14 EPB41L4B MAN1A1 CXorf66 ASXL3 DPF3 CD302 STARD8 NID1 FOXP2 UGT2A2 ACMSD ACOT1 ZG16 ECM2 FAT4 PTCHD3 EBI3 PDE2A ADH1B ANK2 ARHGEF26 PAGE4 SARDH ZDHHC8 CD1D SHE SLC10A1 OR11H6 PTPRD B3GAT1 SCARA5 PLIN4 LRRTM4 OR1E1 F5 ASPA MPZ RFPL4A RANBP3L BTNL9 CRYAA ACSL6 CYP2F1 LIPJ WFIKKN1 HS3ST3B1 KCNB1 SFXN1 EDNRB TRPC5 LIMS2 MAN1C1 PIK3R1 FCN3 ACACB CDO1 SLC23A2 MAF RNF125 A2M MYCT1 SLC46A3 TET1 SEPP1 SDPR CES5A LRP1B HSD17B3 TEK DLC1 SPATA22 PPP1R1A TPTE2 AASS RPGRIP1 CECR2 FBXO27 TACC1 ADHFE1 CCBE1 DCDC5 EIF5 GPX3 GYS2 FXYD1 FAM150B FMO3 SORBS1 PPP1R3C PCDH20 CD5L MASP1 GHR C8B ASTN1 CLEC1B ABCA6 GBP7 GFRA1 MIA3 LIFR GNAT2 ZMAT1 MYL3 APOC3 FAM65C GARNL3 G6PC MYLK ZNF546 SLC16A2 SLC16A12 PRAMEF15 ATP1B2 TRAPPC8 ST6GAL1 ALDH2 RAB37 GABARAPL1 GREB1 LYVE1 ESR1 MT1A MMRN1 PON1 RUNDC3B SOBP TMED5 CFL2 AGTR1 PRSS45 PLCXD3 FNDC5 SERPINA11 ZNF662 CYP27A1 PLIN2 SERPINC1 SLITRK3 CYP2A13 BCHE RHOB AGL CPEB3 SLC7A9 IL1RAP CDHR3 JAKMIP2 EFCAB1 PALMD AKR1D1 CRIP3 CADM2 FAM198A CD36 CYP8B1 ORAI3 MTMR7 PHYHIPL CXCL12 NEU4 DMGDH TTPA NTN1 ADRB2 ATP11C GPC6 TF C19orf12 FZD4 LGI1 SLC6A16 GOLGA6A KANK4 ZIM2 PCP4L1 UNC93A ANKRD55 ITPR2 MPPED1 A1BG CYP4A11 SYPL2 OR4D10 IDO2 AGXT2 PID1 CPN2 CYP1A1 C1QTNF9 PTH1R ART4 AUTS2 TNFSF14 DPYD LY6G6F PHYHD1 VIP SYT9 C7 GLYCTK HYDIN FIGN NCAM1 TMEM56 LRRC7 IL6ST CHST7 HAL ABHD6 SLCO4C1 AQP4 PDZRN4 FREM2 DBH PPP4R4 TBXA2R BMPER ZP4 L2HGDH HSD17B6 HIF1A EHHADH HEPACAM SLC2A4 CES1 WNK3 HGF DTX1 KRT222 IL27 GLYAT OLFM1 ENPP3 COLEC11 ADH1A C11orf54 SCN9A TMEM176B CETP SLIT1 GRIK3 SLC17A2 ISPD C6 METTL7A AFP ANGPTL1 ABCG5 GPAM PAH EFCAB6 KLRB1 MRO RNF165 ZCCHC24 CERS4 CCDC13 PDE3B IP6K3 ACSM5 SLC4A10 FCN2 ACAD11 PLIN1 PRSS42 AFF3 TDRD6 KLKB1 KLRF1 GNAO1 BAI3 LOC285095 LOC100131608 LOC100508384 XLOC_007221 LOC100505976 XLOC_001070 LOC100507203 XLOC_l2_000920 IL28RA LOC388630 LOC100506974 PRAMEF10 LOC100506328 LOC100130503 C2orf77 HEATR7B1 C14orf41 C18orf62 LOC253805 C10orf140 LPAPGCP ABCC9 APOA4 LOC100508383 XLOC_011181 C8orf68 P39189 EFHA2 XLOC_l2_009292 C20orf132 XLOC_l2_013436 XLOC_007093 LOC280665 C9orf95 Q8IWW8 LOC100130232 C5orf4 XLOC_010897 C7orf58 C6orf225 FAM82A1 ODZ1 PZP XLOC_l2_015239

Supplementary Table 4. The 72 DEGs in cluster1 were analyzed by different algorithms of cytoHubba plug-in

Name	MCC	DMNC	MNC	Degree	EPC	Bottle neck	Eccen tricity	Close ness	Radial ity	Between ness	Stress	Clustering Coefficient
KIFC1	9.22E+13	1.555	58	58	13.41	1	0.5	64.5	1.859	4.554	212	0.936
SGOL1	9.22E+13	1.545	53	53	14.458	1	0.5	62	1.789	2.469	118	0.957
CDKN3	9.22E+13	1.588	62	62	15.284	1	0.5	66.5	1.915	5.184	242	0.936
FOXM1	9.22E+13	1.596	65	65	15.113	1	0.5	68	1.958	6.439	304	0.927
CDC25C	9.22E+13	1.587	65	65	15.7	1	0.5	68	1.958	7.017	328	0.921
ARHGAP11A	9.22E+13	1.547	55	55	13.249	1	0.5	63	1.817	3.376	158	0.947
MAD2L1	9.22E+13	1.574	68	68	15.077	1	0.5	69.5	2.000	10.071	452	0.901
CCNA2	9.22E+13	1.575	71	71	16.07	1	0.5	71	2.042	12.277	550	0.889
BIRC5	9.22E+13	1.578	70	70	16.537	1	0.5	70.5	2.028	11.292	508	0.895
CDCA8	9.22E+13	1.575	71	71	16.341	3	1	71	2.042	12.277	550	0.889
MYBL2	9.22E+13	1.563	56	56	14.15	1	0.5	63.5	1.831	3.218	150	0.951
FANCI	9.22E+13	1.581	66	66	15.631	1	0.5	68.5	1.972	8.169	372	0.913
NEK2	9.22E+13	1.584	66	66	15.301	1	0.5	68.5	1.972	7.993	364	0.915
TPX2	9.22E+13	1.575	71	71	16.277	1	1	71	2.042	12.277	550	0.889
MCM4	9.22E+13	1.583	64	64	15.382	1	0.5	67.5	1.944	6.700	308	0.924
CDT1	9.22E+13	1.587	65	65	15.16	1	0.5	68	1.958	7.093	326	0.922
ATAD2	9.22E+13	1.519	50	50	12.393	1	0.5	60.5	1.746	2.183	102	0.958
AURKA	9.22E+13	1.575	70	70	16.538	1	0.5	70.5	2.028	11.462	514	0.894
CCNB2	9.22E+13	1.575	71	71	16.207	1	1	71	2.042	12.277	550	0.889
BUB1B	9.22E+13	1.575	71	71	16.526	1	1	71	2.042	12.277	550	0.889
RAD54L	9.22E+13	1.592	63	63	15.433	1	0.5	67	1.930	5.599	260	0.933
CDK1	9.22E+13	1.575	71	71	15.495	2	1	71	2.042	12.277	550	0.889
CEP55	9.22E+13	1.581	68	68	15.727	1	0.5	69.5	2.000	9.531	434	0.905

CENPM	9.22E+13	1.591	57	57	14.072	1	0.5	64	1.845	2.453	118	0.963
CCNB1	9.22E+13	1.575	71	71	16.252	1	1	71	2.042	12.277	550	0.889
POLQ	9.22E+13	1.562	52	52	13.061	1	0.5	61.5	1.775	1.4777	70	0.974
PTTG1	9.22E+13	1.586	66	66	15.832	1	0.5	68.5	1.972	7.792	358	0.917
CDCA7	9.22E+13	1.516	47	47	12.412	1	0.5	59	1.704	1.094	52	0.976
PRC1	9.22E+13	1.580	68	68	15.368	1	0.5	69.5	2.000	9.616	436	0.904
GTSE1	9.22E+13	1.599	59	59	15.133	1	0.5	65	1.873	2.992	146	0.957
PLK1	9.22E+13	1.580	68	68	15.607	1	0.5	69.5	2.000	9.653	436	0.904
FAM64A	9.22E+13	1.585	55	55	13.873	1	0.5	63	1.817	1.770	88	0.970
CDC20	9.22E+13	1.578	70	70	16.203	1	0.5	70.5	2.028	11.292	508	0.895
KIF20B	9.22E+13	1.523	53	53	14.349	1	0.5	62	1.789	3.330	156	0.943
RFC3	9.22E+13	1.535	51	51	13.731	1	0.5	61	1.761	2.022	96	0.962
HELLS	9.22E+13	1.518	53	53	13.506	1	0.5	70.5	1.789	3.591	164	0.940
MKI67	9.22E+13	1.577	70	70	16.47	1	0.5	65	2.028	11.344	510	0.894
BRCA1	9.22E+13	1.561	59	59	14.032	1	0.5	65	1.873	4.856	224	0.935
KIF18B	9.22E+13	1.542	55	55	13.142	1	0.5	63	1.817	3.616	166	0.944
ASPM	9.22E+13	1.575	71	71	15.792	1	1	71	2.042	12.277	550	0.889
KIAA0101	9.22E+13	1.569	67	67	15.781	1	0.5	69	1.986	9.622	432	0.902
UBE2T	9.22E+13	1.559	52	52	13.334	1	0.5	61.5	1.775	1.575	76	0.971
RRM2	9.22E+13	1.575	70	70	15.281	1	0.5	70.5	2.028	11.482	514	0.894
CHEK1	9.22E+13	1.574	66	66	15.003	1	0.5	68.5	1.972	8.632	388	0.910
CCNF	9.22E+13	1.533	49	49	12.634	1	0.5	60	1.732	1.308	62	0.974
ASF1B	9.22E+13	1.586	64	64	15.286	1	0.5	67.5	1.944	6.535	300	0.926
KIF14	9.22E+13	1.584	60	60	14.4	1	0.5	65.5	1.887	4.271	200	0.944
CENPF	9.22E+13	1.575	71	71	16.433	1	1	71	2.042	12.277	550	0.889

DTL	9.22E+13	1.577	68	68	15.563	1	0.5	69.5	2.000	9.850	444	0.903
FEN1	9.22E+13	1.580	61	61	15.349	1	0.5	66	1.901	5.110	234	0.936
TROAP	9.22E+13	1.573	55	55	13.603	1	1	63	1.817	2.291	110	0.963
KIF11	9.22E+13	1.575	71	71	15.945	1	0.5	71	2.042	12.277	550	0.889
CASC5	9.22E+13	1.535	50	50	13.342	1	0.5	60.5	1.746	1.607	76	0.969
ESCO2	9.22E+13	1.505	49	49	12.57	1	0.5	60	1.732	2.298	104	0.956
KIF4A	9.22E+13	1.580	70	70	15.514	1	0.5	70.5	2.028	11.081	502	0.896
EXO1	9.22E+13	1.578	68	68	15.205	1	0.5	69.5	2.000	9.781	442	0.903
ZWINT	9.22E+13	1.580	66	66	14.918	1	0.5	68.5	1.972	8.291	376	0.912
MCM7	9.22E+13	1.556	58	58	14.309	1	0.5	64.5	1.859	4.538	210	0.936
CLSPN	9.22E+13	1.545	55	55	13.844	1	0.5	63	1.817	3.383	160	0.946
DLGAP5	9.22E+13	1.575	71	71	16.155	1	1	71	2.042	12.277	550	0.889
KIF2C	9.22E+13	1.575	71	71	15.95	1	1	71	2.042	12.277	550	0.889
KIF23	9.22E+13	1.575	71	71	16.107	1	1	71	2.042	12.277	550	0.889
STIL	9.22E+13	1.508	46	46	11.847	1	0.5	58.5	1.690	0.975	46	0.978
TOP2A	9.22E+13	1.575	71	71	15.569	1	1	71	2.042	12.277	550	0.889
AURKB	9.22E+13	1.575	71	71	16.006	1	1	71	2.042	12.277	550	0.889
KIF22	9.22E+13	1.549	52	52	13.561	1	0.5	61.5	1.775	1.894	92	0.965
UBE2C	9.22E+13	1.578	70	70	15.784	1	0.5	70.5	2.028	11.236	506	0.895
MELK	9.22E+13	1.575	71	71	16.13	1	1	71	2.042	12.277	550	0.889
HMMR	9.22E+13	1.572	70	70	15.978	1	0.5	70.5	2.028	11.727	524	0.892
CDC45	9.22E+13	1.575	71	71	16.382	1	1	71	2.042	12.277	550	0.889
KIF20A	9.22E+13	1.575	71	71	16.281	1	1	71	2.042	12.277	550	0.889
NUSAP1	9.22E+13	1.575	71	71	16.618	1	1	71	2.042	12.277	550	0.889

Supplementary Table 5. PLK1 mRNA expression in gallbladder carcinoma and adjacent tissues

Group	Case	PLK1 mRNA(Δ Ct)	$2^{-\Delta\Delta Ct}$	P value
GBC tissue	15	5.82±1.54	1.67	0.006
Adjacent tissue	15	6.56±1.87		

Supplementary Table 6. CEP55 mRNA expression in gallbladder carcinoma and adjacent tissues

Group	Case	CEP55 mRNA(Δ Ct)	$2^{-\Delta\Delta Ct}$	P value
GBC tissue	15	4.24±1.62	2.969	<0.001
Adjacent tissue	15	5.81±1.83		

Supplementary Table 7. Relationship between expression of CEP55 protein and survival time in gallbladder carcinoma

CEP55 expression	Cumulative survival situation		
	1-year	2-year	3-year
High	31.57%	17.03%	11.17%
Low	59.41%	34.39%	23.32%
